# From routine to selective: how updated MRI guidelines reshape gadolinium use in Germany

**DOI:** 10.1186/s42466-025-00387-y

**Published:** 2025-05-19

**Authors:** Marc Pawlitzki, Alexander Stahmann, Niklas Frahm, Mathia Kirstein, Melanie Peters, Peter Flachenecker, Tim Friede, Kerstin Hellwig, Dagmar Krefting, Michaela Mai, Clemens Warnke, Uwe K. Zettl, David Ellenberger

**Affiliations:** 1 Department of Neurology, Medical Faculty, University Hospital Düsseldorf, Düsseldorf, Germany; 2 German MS Register, MS Forschungs- und Projektentwicklungs- gGmbH (MS Research and Project Development gGmbH [MSFP]), Hannover, Germany; 3 German MS Register, Gesellschaft für Versorgungsforschung mbH (Society for Health Care Research [GfV]), Hannover, Germany; 4 Neurological Rehabilitation Center Quellenhof, Bad Wildbad, Germany; 5 Department of Medical Statistics, University Medical Center Göttingen, Göttingen, Germany; 6 Department of Neurology, St. Josef Hospital, Ruhr University, Bochum, Germany; 7 Department of Medical Informatics, University Medical Center Göttingen, Göttingen, Germany; 8 Deutsche Multiple Sklerose Gesellschaft, Bundesverband e.V. (German Multiple Sclerosis Society [DMSG]), Hannover, Germany; 9 Department of Neurology, University Hospital of Gießen and Marburg, Marburg, Germany; 10 Department of Neurology, Neuroimmunological Section, University Medical Center of Rostock, Rostock, Germany

**Keywords:** Relapsing multiple sclerosis, Magnetic resonance imaging, Gadolinium-based contrast

## Abstract

Magnetic resonance imaging (MRI) is a critical diagnostic tool and monitoring modality for multiple sclerosis (MS), frequently employing gadolinium-based contrast agents (Gd). However, concerns regarding the accumulation of Gd have prompted international guidelines (MAGNIMS-CMSC-NAIMS, 2021) to advocate for the limitation of Gd utilization. Consequently, we assessed of the impact of the 2021 guidelines on the use of Gd in MRI in MS patients in Germany by conducting a retrospective analysis of MRI data from 12,833 MS patients in the German MS Register (2019–2024). Generalized additive models were employed to analyze Gd use trends over time by MRI type (cranial, spinal, combined). From 2020 to 2024, a significant decline in Gd use was observed, with percentages dropping from 74.2 to 41.2% in cranial MRI, from 78.2 to 39.2% in spinal MRI and from 81.8 to 59.0% in combined MRI (*p* < 0.001). The most substantial decline occurred within the initial five years of MS. Gd use in MS MRI scans has significantly decreased in line with the updated guidelines. Nevertheless, its persistent utilization in over one-third of cases necessitates further examination.

Magnetic resonance imaging (MRI) remains central to diagnosing and monitoring multiple sclerosis (MS). Traditionally, gadolinium-based contrast agents (Gd) have been widely used to detect active inflammatory lesions, aiding in treatment decisions [1]. However, concerns about Gd accumulation and potential side effects [2] have prompted revisions to international guidelines [3].

In 2021, the MAGNIMS-CMSC-NAIMS consensus recommendations updated the guidelines on Gd use, advising it only in cases where clear additional benefit is expected, such as when confirming an MS diagnosis or if no suitable reference MRI is available [4]. This change reflects the need to balance diagnostic accuracy with patient safety, emphasizing a more cautious approach to Gd use in MS management. However, there is currently limited understanding of how effectively these updated guidelines have been implemented in clinical practice.

Our retrospective study utilized MRI data from 12,833 MS patients in the German MS Register (GMSR), encompassing 23,934 MRI scans. We employed generalized additive models to evaluate Gd use trends by MRI type (cranial, spinal, combined). The findings revealed a marked reduction in Gd administration: cranial MRI (74.2% in 2020 to 41.2% in 2024, *p* < 0.001), spinal MRI (78.2–39.2%, *p* < 0.001) and combined MRI (81.8–59.0%, *p* < 0.001). Notably, the most significant decline was observed within the first five years of disease onset, suggesting an increasing tendency to restrict Gd use to select clinical scenarios rather than routine follow-up scans (Fig. 1).

**Fig. 1:**
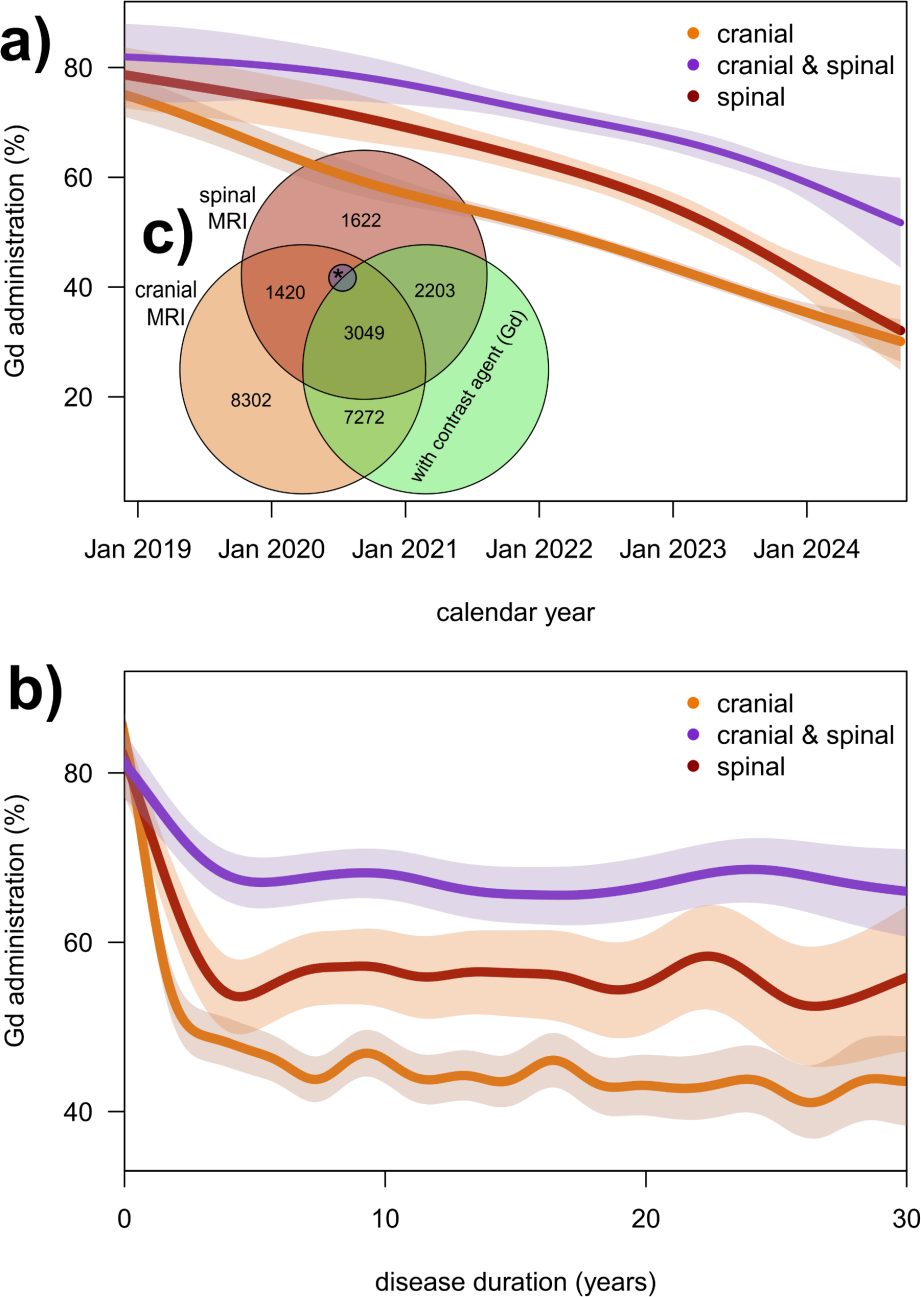
Contrast agent use in MRI: Analysis by MRI type, location, year, and disease duration since 2019. **(a)** Frequency of Gd administration in MRI examinations by type/location of examination and calendar year. The shaded areas represent the associated 95% confidence intervals. **(b)** Frequency of contrast agent administration in MRI examinations broken down by type/location of examination and duration of disease. The shaded areas represent the corresponding 95% confidence intervals. **(c)** Venn diagram of MRI examinations since January 1, 2019 by type: cranial vs. spinal separated by with vs. without contrast agent administration. *There are 61 MRI examinations in which both a cranial and a spinal MRI were performed, but Gd was only used in one type of MRI (Gd may have been given between both MRI). These rare cases were excluded from further analyses. Gd– Gadolinium-based contrast agents; MRI– Magnetic Resonance Imaging

These results underscore the rapid implementation of guideline-driven changes in clinical practice. The observed decrease aligns with an emerging consensus to limit unnecessary Gd exposure while maintaining diagnostic accuracy [4]. However, despite this reduction, Gd was still used in over one-third of MRI scans in early 2024. This raises important questions regarding the ongoing necessity of contrast-enhanced imaging in certain cases. Possible explanations include the need to confirm active disease in complex cases, such as progressive MS or inconclusive imaging findings [5]. However, the assumption that the administration of Gd-based agents indicates an acute clinical event has recently been increasingly questioned, as many MS-specific disease progressions are not captured by conventional imaging methods [6].

Emerging evidence suggests that alternative MRI markers, such as the central vein sign and paramagnetic rim lesions, may provide comparable or superior insights into MS activity and progression [7]. Integrating these biomarkers into routine imaging protocols may further reduce reliance on Gd while preserving diagnostic accuracy [7]. However, prioritizing imaging sequences for these markers in daily routine seems to be a current challenge.

The variability in Gd-based contrast use among MS centers, influenced by institutional preferences or patient factors (e.g., high numbers of newly diagnosed cases), reflects inconsistencies with guidelines. A European survey supports this, showing routine Gd use during follow-ups in over 30% of institutions [8]. A more granular analysis of the clinical indications prompting contrast administration could help clarify whether deviations from guidelines are justified. It is crucial to determine whether continued Gd use primarily occurs in newly diagnosed patients requiring baseline scans, or in patients with ambiguous imaging findings like radiologically isolated syndrome findings requiring enhanced visualization [9]. Moreover, the discrepancy in Gd use may also reflect differing perceptions among clinicians regarding its necessity for evaluating disease progression or distinguishing pseudo-progression from true relapse. Understanding these patterns may inform future refinements to guideline recommendations and standardized imaging protocols across healthcare settings.

Additionally, patient perspectives on Gd administration warrant consideration. MS patients might express concerns about repeated contrast exposure, particularly in light of reports of Gd retention in brain tissue [3]. Enhanced patient education regarding the rationale behind reducing Gd use may facilitate shared decision-making and improve adherence to updated imaging protocols.

While our data do not fully capture the broader impact of this shift in clinical practice, future research should also clarify the specific consequences of decreasing Gd use in case of MS. Understanding whether reduced Gd administration compromises the diagnostic accuracy especially in terms of radiologically isolated syndrome [9], the detection of subclinical disease activity or influences therapeutic strategies is essential to maintaining disease monitoring and optimizing patient outcomes. Interestingly, emerging fluid biomarkers such as neurofilament light chain in sera, which serves as a marker of acute MS-related inflammation in the brain and spinal cord, could further refine the selective use of Gd by providing additional insights into disease activity and progression [10]. To further elucidate the clinical implications of the guideline adoption, future investigations should assess the following aspects: (1) the proportion of patients whose diagnoses are revised (e.g. tumour, neurosarcoidosis, vasculitis) following subsequent imaging with Gd administration; (2) the frequency of misdiagnoses or diagnostic delays attributable to the reduced use of Gd; and (3) the impact of the guidelines on therapeutic outcomes, including changes in disease activity, disability progression and relapse rates. Such analyses will yield critical insights into the potential consequences of the revised guidelines on patient outcomes and treatment strategies.

## Data Availability

Anonymized data will be made available on request for any qualified investigator under the terms of the registry’s usage and access guidelines and subject to the informed consent of the patients.

## References

[1] Lindland, E. S. (2023). Using contrast-medium administration in multiple sclerosis: we need much more confidence than the chance of seeing a shooting star when looking up at the night sky. *Eur Radiol*, *34*, 1724–1725. 10.1007/s00330-023-10272-4 10.1007/s00330-023-10272-4 37782340

[2] Gulani, V., Calamante, F., Shellock, F. G., Kanal, E., & Reeder, S. (2017). B. Gadolinium deposition in the brain: Summary of evidence and recommendations. *Lancet Neurol*, *16*, 564–570. 28653648 10.1016/S1474-4422(17)30158-8

[3] van der Molen, A. J., Quattrocchi, C. C., Mallio, C. A., & Dekkers, I. A. & for the European society of magnetic resonance in medicine, biology gadolinium research, educational committee (ESMRMB-GREC). (2023). Ten years of gadolinium retention and deposition: ESMRMB-GREC looks backward and forward. *Eur Radiol*, *34*, 600–611. 37804341 10.1007/s00330-023-10281-3 PMC10791848

[4] Wattjes, M. P., et al. (2021). 2021 MAGNIMS–CMSC–NAIMS consensus recommendations on the use of MRI in patients with multiple sclerosis. *Lancet Neurol*, *20*, 653–670. 34139157 10.1016/S1474-4422(21)00095-8

[5] Rovira, À., et al. (2023). Use of gadolinium-based contrast agents in multiple sclerosis: A review by the ESMRMB-GREC and ESNR multiple sclerosis working group. *Eur Radiol*, *34*, 1726–1735. 37658891 10.1007/s00330-023-10151-y

[6] Gavoille, A., et al. (2024). Acute clinical events identified as relapses with stable magnetic resonance imaging in multiple sclerosis. *JAMA Neurol*, *81*, 814. 38949816 10.1001/jamaneurol.2024.1961 PMC11217890

[7] Borrelli, S., et al. (2024). Central vein sign, cortical lesions, and paramagnetic rim lesions for the diagnostic and prognostic workup of multiple sclerosis. *Neurol Neuroimmunol Neuroinflammation*, *11*, e200253. 10.1212/NXI.0000000000200253 PMC11129678 38788180

[8] Hodel, J., et al. (2023). Multiple sclerosis imaging in clinical practice: A European-wide survey of 428 centers and conclusions by the ESNR working group. *Eur Radiol*, *33*, 7025–7033. 37199796 10.1007/s00330-023-09701-1

[9] Okuda, D. T., & Lebrun-Frénay, C. (2024). Radiologically isolated syndrome in the spectrum of multiple sclerosis. *Mult Scler*, *30*, 630–636. 38619142 10.1177/13524585241245306 PMC11071642

[10] Freedman, M. S., et al. (2024). Guidance for use of neurofilament light chain as a cerebrospinal fluid and blood biomarker in multiple sclerosis management. *EBioMedicine*, *101*, 104970. 38354532 10.1016/j.ebiom.2024.104970 PMC10875256

